# Education Does Not Affect Cognitive Decline in Aging: A Bayesian Assessment of the Association Between Education and Change in Cognitive Performance

**DOI:** 10.3389/fpsyg.2018.01138

**Published:** 2018-07-06

**Authors:** Rasmus Berggren, Jonna Nilsson, Martin Lövdén

**Affiliations:** Aging Research Center, Karolinska Institutet, Stockholm University, Stockholm, Sweden

**Keywords:** cognitive aging, Bayesian inference, cognitive reserve, longitudinal study, cohort study

## Abstract

Education is positively associated with level of cognitive function but the association between education and rate of cognitive decline remains unresolved, partly for methodological reasons. In this article, we address this issue using linear mixed models and Bayesian hypothesis testing, using data from the Betula cohort-sequential longitudinal study. Our results support the null hypothesis that education does not alter the rate of cognitive decline for visuospatial ability, semantic knowledge, and episodic memory. We propose that education is only a relevant variable for understanding cognitive performance in older age because of the association between performance and education that is formed in early development.

## Introduction

Cognitive performance declines in aging. Longitudinal studies estimate that average decline in reasoning performance starts already in middle age (Schaie, 1994, 2005; Rönnlund and Nilsson, 2006) whereas knowledge-based performance increases through middle age, but then also decreases in older age (Schaie, 1994, 2005; Rönnlund et al., 2005). Between-person differences in both level and within-person change of cognitive performance are however large, especially in older age (de Frias et al., 2007). Formal education is a potential predictor of these between-person differences that has received much attention, but no agreement has been reached in regards to the role that education plays in cognitive aging (Anstey and Christensen, 2000; Glymour et al., 2005; Valenzuela and Sachdev, 2006; Stern, 2009; Tucker-Drob et al., 2009; Deary and Johnson, 2010; Tucker and Stern, 2011; Meng and D'Arcy, 2012; Dekhtyar et al., 2015; Lenehan et al., 2015).

Assuming that cognitive aging can be described by level of performance in early adulthood and the change thereafter (Hertzog, 1985), there are two possible ways in which education could relate to cognitive aging: Educational attainment could predict level of performance or change in performance. Both effects may of course also be present. Importantly, influences of education on level of performance may have important implication for individual cognitive aging even in the absence of effects on change, as a higher level of performance may postpone the age at which functional impairment thresholds are reached (e.g., Satz, 1993; Lövdén et al., 2010).

Education, either operationally defined as highest achieved degree or years of education after entry to primary education, has a solid association with level of cognitive performance among adults, and this association persists into old age (Strenze, 2007; Opdebeeck et al., 2015). Meta-analyses show that the magnitude of this association is around *r* = 0.3 (uncorrected for reliability) for typical neuropsychological tests administered to older adults (Opdebeeck et al., 2015).

Whilst the positive association between educational attainment and level of cognitive function is well established, the exact nature of this relationship is complex. Part of this association may stem from causal effects of education (Baltes and Reinert, 1969; Ceci, 1991; Cliffordson and Gustafsson, 2008; Lager et al., 2017), such that education improves cognitive function. Another possibility is that people with higher cognitive ability seek and gain access to longer education. Yet another possibility, which is perhaps the most plausible one, is that there is a complex interplay between cognitive function and educational attainment. For example, individuals with higher innate ability may stay in school longer, which in turn has beneficial effects on cognitive function in adulthood.

The magnitude of the association between education and level of cognitive function may also be shaped by a range of factors, and even change across time. The relationship between education and cognitive function could for example vary with societal differences and with historical changes that influence birth cohorts differently (Rönnlund and Nilsson, 2009). The substantial increases of education during the twentieth century and in particular the accompanying increases of socioeconomic equality of educational opportunities in some countries could be one source of such variation (Johnson et al., 2010; Branigan et al., 2013). For example, Heath et al. (1985) reported increased heritability of education for Norwegian men born between 1940 and 1949 as compared to men born before 1940, suggesting a reduced dependency of education attainment on socioeconomic environment and an increased dependency on innate ability in later birth cohorts. Thus, cognitive ability in childhood could be a stronger predictor of educational attainment in a more meritocratic society. Working under the assumption that a society has progressed toward such a society during the twentieth century, this would imply that the association between level of cognitive function and educational attainment is stronger in later born cohorts.

In contrast to the robust relationship between educational attainment and level of cognitive function, the relationship between educational attainment and age-related change in cognitive performance is less clear. There are three possibilities regarding this relationship. First, higher education may increase the capacity to counteract negative brain changes in aging (e.g., through compensatory strategies) and individuals with higher education may therefore exhibit a *slower* rate of cognitive decline. This hypothesis sometimes goes under the name of an “active reserve” model (see e.g., Stern, 2009; Barulli and Stern, 2013). Second, education may delay the onset of decline by making use of auxiliary brain structures once core brain structures begin to deteriorate, but that the rate of decline should be *faster* once these auxiliary brain structures deteriorate too. This hypothesis has been termed the “neural compensation hypothesis” (see e.g., Lenehan et al., 2015). Third, education may only alter the level of cognitive function but not alter the rate of decline thereafter. This hypothesis is sometimes summarized under “passive reserve” models (see e.g., Stern, 2009; Barulli and Stern, 2013).

The empirical results do not unanimously support either hypothesis: whilst some studies have reported positive associations (Arbuckle et al., 1998; Lyketsos et al., 1999; Lipnicki et al., 2017), others have reported negative associations (Alley et al., 2007; Hülür et al., 2013; Gross et al., 2015) or no statistically significant associations at all (e.g., Tucker-Drob et al., 2009; Gerstorf et al., 2011; Zahodne et al., 2011) between education and cognitive change in adulthood and aging. Lenehan et al. (2015) also suggest that later studies and studies using more sophisticated methodological approaches found no (statistically significant) association between educational attainment and rate of cognitive decline. Thus, the dissemination and use of more advanced statistical techniques for analyzing longitudinal data in recent years may partly explain this discrepancy between earlier and later reports (Glymour et al., 2005; Zahodne et al., 2011; Lenehan et al., 2015).

Evidently, the three models introduced above make different predictions regarding the relationship between education and rate of cognitive decline. Importantly, one of the models—that of passive reserve—predicts that education does not alter the rate of cognitive decline, which coincides with the statistical null hypothesis of invariance. It is therefore unfortunate that prior work utilize significance testing of parameter estimates, which is unable to quantify evidence in favor of the null hypothesis. To address this hypothesis directly, we here instead employ Bayesian hypothesis testing using Bayes factors, which allows us to quantify the evidence in favor of the null hypothesis. The data come from the Betula cohort-sequential study (Nilsson et al., 1997, 2004) and we use it to investigate the influence of education on level of cognitive performance (spatial reasoning, semantic knowledge, and episodic memory) and within-person change in performance, and to explore the moderating influences of birth cohort.

## Methods

The data was obtained from the steering group of the Betula study^1^. The design and procedures of the Betula longitudinal study has been described in detail elsewhere (Nilsson et al., 1997, 2004). Here, we describe methods immediately relevant for the present study.

### Participants

Written informed consent were obtained from all participants at study inclusion. The first wave of data collection took place between 1988 and 1990, during which 100 participants from the age groups of 35, 40, …, 80 years (i.e., born 1953–1955, 1948–1950, …, 1908–1910) were randomly sampled from the population registry of Umeå in northern Sweden (Sample 1; S1). Only participants free from a dementia diagnosis were eligible for entry to the study. Participants that were willing and able to come back for repeated testing were followed up every fifth year over 20 years (at 1993–1995, 1998–2000, 2003–2005, and 2008–2010). At each new assessment, another age-matched sample were included in the study. Here we used the original sample (S1) and sample 3 (S3), which was included in the study at the assessment in 1993–1995 (Time 2; T2) and was then consisting of 100 participants from the age groups of 35, 40, …, 85 years (i.e., born 1958–1960, 1953–1955, …, 1908–1910). S3 has also been fully followed up every fifth year, until the 2008–2010 data collection wave. Previous analyses have found that the samples are representative of the target population in Umeå (Nilsson et al., 1997). For the analyses, we excluded the earliest (1908–1910) and latest born cohorts (1958–1960), because data density was low in these design cells.

### Procedures and measures

Cognitive data were collected during two sessions by a nurse or by a trained psychologist (see Nilsson et al., 1997, for details). Education was operationally defined as self-reported years of education and was represented by the maximum number of years reported over the five assessments. As dependent variables, we selected one measure of visuospatial ability (VA), one measure of episodic memory (EM), and one measure of semantic knowledge (SK) that had good psychometric properties across the age-range of the sample (i.e., acceptable reliability and absence of ceiling and floor effects).

Visuospatial ability was assessed using the standard block design task in WAIS-R. Performance on this task correlates strongly with measures of general intelligence (Wechsler, 1981; Ryan et al., 1990). The participants were given a set of 4 or 9 cubic blocks, and was asked to arrange the blocks to re-create patterns shown to them on paper, with a maximum of 10 patterns. The test was administered and scored in accordance with the WAIS-R manual (Wechsler, 1981).

To measure episodic memory performance, participants were presented with 16 verbal instructions involving a verb and a noun (e.g., “lift the book,” “point at the pencil”) that were performed by the participant. Participants was also told that they would later recall the instructions. Immediately afterwards, the participant was asked to recall out loud as many of the instructions as possible. The outcome measure was the number of instructions correctly recalled (both correct verb *and* noun) of the sentences.

For the measure of semantic knowledge, participants were presented with a 30-item multiple choice synonym test (Dureman, 1960). The task was to pick out the correct synonym to the target word among five options. Participants were given a time-limit of 7 min for all 30 items. The dependent variable was the number of correct choices.

We used all available data in the analyses (i.e., no listwise deletion), with exception of excluding 14 subjects due to missing data on education, 4 subjects because they reported >25 years of education, and 24 subjects due to reporting less than compulsory schooling. The total numbers of participants used in the analyses (i.e., participants with at least one score at any of the assessments) were 1,707 for visuospatial ability and episodic memory, (54% female) and 1,697 for semantic knowledge (54% female).

### Statistical procedures

#### Data preparation

Age, cohort and education was mean-centered at ~62.9 years of age; born in 1933; and having an average of 10.2 years of education, respectively. Age was further decomposed into a linear term *age* and an orthogonal quadratic term *age*^2^, in order to capture both linear (rate of decline) and quadratic trends (rate of acceleration). In line with previous results from the Betula study (e.g., Rönnlund and Nilsson, 2008), cohort was coded as a linear effect. Sex was coded as 0 for males, 1 for females. All three outcome measures were T-standardized to have a mean of 50 and a standard deviation of 10, based on the mean and SD of the respective test at first measurement occasion (T1). For our purposes, T-standardization facilitates the formulation of priors because the literature we base them on uses T-scores.

#### Model specification

In order to account for the repeated measurements and missing data, we used a linear mixed modeling approach. The model was set up following the general framework for cohort-sequential data described by Galbraith et al. (2017; Model 7; see also Gerstorf et al., 2011). This model allows us to model age-based changes and cohort effects simultaneously, albeit under the assumption of no period effects. The assumption of no period effects is necessary for the model to be identified, because there is a perfect linear dependency between age, cohort and period (e.g., age = cohort + period; Bell and Jones, 2013).

Since age, in our longitudinal design, is a within-subject variable, the linear mixed approach allows us estimate both fixed (average) intercepts and slopes as well as random (subject-specific) intercepts and slopes. Cohort, education, and sex were modeled as fixed effects only and were allowed to interact with age and with each other. Sex is included as a covariate to partition out sex differences. Because subjects of a particular age and a particular birth cohort enter the study at different time points (e.g., S1 and S3), these will differ only with respect to their testing experience. We therefore included sample in the model, as a main effect only, in an attempt to capture some of the potential test-retest effects.

The model was estimated separately for visuospatial ability, semantic knowledge and episodic memory. Using multilevel notation, we model a person *i*'s cognitive ability *Y* at age *t*, *Y*_*ti*_, with an intercept term β_0*i*_, a linear slope term β_1*i*_, a (orthogonal) quadratic slope term β_2*i*_ plus residual error ε_*ti*_. We specify the Level 1 model as

$$Y_{ti}=\;\beta_{0i}+\beta_{1i}\;(age_{ti})+\beta_{2i}(age_{ti}^{2})+\;\varepsilon_{ti}$$

The Level 2 model is specified as

$$\begin{array}{l} \beta_{0i}=\;\gamma_{00}+\;\gamma_{01}\left(\mathrm{cohor}t_{i}\right)+\;\gamma_{02}\left(\mathrm{educatio}n_{i}\right)+\;\gamma_{03}\left(\mathrm{se}x_{i}\right)\\ \;+\;\gamma_{04}\left(\mathrm{sampl}e_{i}\right)+\;\gamma_{05}\left(\mathrm{cohor}t_{i}\;\times \;\mathrm{educatio}n_{i}\right)\\ \;+\;\gamma_{06}\left(\mathrm{cohor}t_{i}\times \mathrm{se}x_{i}\right)+\;\gamma_{07}\left(\mathrm{educatio}n_{i}\times \mathrm{se}x_{i}\right)\\ \;+\;\gamma_{08}\left(\mathrm{cohor}t_{i}\times \mathrm{educatio}n_{i}\times \mathrm{se}x_{i}\right)+\;u_{0i},\\ \beta_{1i}=\;\gamma_{10}+\;\gamma_{11}\left(\mathrm{cohor}t_{i}\right)+\;\gamma_{12}\left(\mathrm{educatio}n_{i}\right)\\ \;+\;\gamma_{13}\left(\mathrm{se}x_{i}\right)+\;\gamma_{14}\left(\mathrm{cohor}t_{i}\;\times \;\mathrm{educatio}n_{i}\right)\\ \;+\;\gamma_{15}\left(\mathrm{cohor}t_{i}\times \mathrm{se}x_{i}\right)+\;\gamma_{16}\left(\mathrm{educatio}n_{i}\times \mathrm{se}x_{i}\right)\\ \;+\;\gamma_{17}\left(\mathrm{cohor}t_{i}\times \mathrm{educatio}n_{i}\times \mathrm{se}x_{i}\right)+\;u_{1i}, \end{array}$$

and

$$\begin{array}{l} \beta_{2i}=\;\gamma_{20}+\;\gamma_{21}\left(\mathrm{cohor}t_{i}\right)+\;\gamma_{22}\left(\mathrm{educatio}n_{i}\right)+\;\gamma_{23}\left(\mathrm{se}x_{i}\right)\\ +\;\gamma_{24}\left(\mathrm{cohor}t_{i}\;\times \;\mathrm{educatio}n_{i}\right)+\;\gamma_{25}\left(\mathrm{cohor}t_{i}\times \mathrm{se}x_{i}\right)\\ +\;\gamma_{26}\left(\mathrm{educatio}n_{i}\times \mathrm{se}x_{i}\right)\\ +\;\gamma_{27}\left(\mathrm{cohor}t_{i}\times \mathrm{educatio}n_{i}\times \mathrm{se}x_{i}\right) \end{array}$$

β_0i_ specifies the initial level of cognitive function, β_1i_ specifies the linear decline (the first derivative, “change”) with advancing age, and β_2i_ specifies the accelerated decline (the second derivative, “change in change”) with advancing age. We further include subject-specific intercepts *u*_0i_ and linear slopes *u*_1*i*_, as well as estimate the correlation between intercept and slope, ρ.

Of focal interest for hypothesis testing is the parameter γ_12_, which captures the effect of educational attainment on cognitive decline. This parameter is tested via Bayes factors, using prior information about the magnitude of that effect from Gerstorf et al. (2011) and Hülür et al. (2013). These articles were selected because both they use a similar longitudinal cohort-sequential design and sample from a similar population (adults born in the early-to-mid twentieth century); similar independent variables (age, cohort, education, sex) and dependent variables (episodic memory, semantic knowledge, visuospatial ability); and similar units of measurement. This makes the formulation of prior distributions for γ_12_ straightforward.

Exploratory analyses investigate the moderating effect of birth cohort on the effect of educational attainment on cognitive function (parameter γ_05_). We opt for an exploratory approach because very little work has been done investigating the differential linear effect of education on level of cognitive function across different cohorts (e.g., if later birth cohorts benefit more or less from education). We explore this parameter through interval estimation. We also relate obtained parameter estimates of linear decline (γ_10_) and educational attainment (γ_02_) to previous findings in the literature.

#### Specification of priors

We utilize a Bayesian approach to hypothesis testing, relying on Bayes factors. For the specification of priors regarding the effect of education on cognitive decline, we consulted Gerstorf et al. (2011) for visuospatial ability (VA) and semantic knowledge (SK), and Hülür et al. (2013) for episodic memory (EM). The corresponding parameter estimates regarding the effect of education on cognitive decline, expressed in T-units difference in cognitive change over a 1 year period per year of education, are −0.002 for VA and −0.004 for SK, and −0.017 for EM. Of note is that Gerstorf et al. (2011) report statistically non-significant effects of education on cognitive decline of VA (β = −0.002, SE = 0.003, *n.s*.) and SK (β = −0.004, SE = 0.003, n.s.) whereas Hülür et al. (2013) report a statistically significant effect of education on cognitive decline of EM (β = −0.017, SE = 0.005, *p* < 0.05), indicating that higher educational attainment is associated with steeper linear decline.

We model the prior for the effect of education on linear decline (i.e., γ_12_) as a normal distribution centered on 0, with a standard deviation σ equal to 1, 2, or 4 times the absolute value of the parameter estimates from Gerstorf et al. (2011) and Hülür et al. (2013). We center the prior distribution on 0 because γ_12_ = 0 is the null hypothesis of interest, and as such constitutes a conservative test of the null hypothesis. The prior γ_12_ ~ Normal(0, σ) captures the alternative hypothesis that smaller (absolute) values are more plausible than large (absolute) values, and that the true parameter value lies between 1.96 σ with probability 0.95. The range of priors (1, 2, 4 σ) also constitute our sensitivity analysis.

For all parameters except γ_12_, we used weakly informative priors. The intercept was modeled with a normal (50, 20) prior; regression parameters were modeled using normal (0, 3) priors; random effects variance parameters were modeled using half-Cauchy (0, 10) priors; correlation between random intercept and slope was modeled using an LKJ (1) (e.g., flat) prior. The choice of weakly informative priors was motivated by the fact that those parameters were not subject to hypothesis testing. Full specification can be found in Supplement A. Bayes factors for γ_12_ was approximated by calculating the ratio of the prior and posterior densities at γ_12_ = 0 (see e.g., Lee and Wagenmakers, 2013).

## Data analysis

The model was estimated using the rstan (Stan Developent Team, 2016) and brms (Bürkner, 2017) packages in R (R Core Team, 2018). Plots were generated using ggplot2 (Wickham, 2009). We sampled 2,000 samples, using 4 parallel chains. All chains indicated convergence, according to the Gelman-Rubin rhat statistic (rhat < 1.01). See Supplement A for further details.

## Results

Parameter estimates for the random effects model, using weakly informative priors for all parameters (including γ_12_) are presented in Table 1. Parameter estimates for γ_12_, the focal parameter that captures the effect of educational attainment on (linear) cognitive decline, differ only marginally under different priors (see Table 2). We report maximum a posterior estimate (MAP) as well as 95% highest density intervals (HDI) and, for γ_12_, Bayes factors under different priors.

**Table 1:** Estimates from the full random effects model using weakly informative priors.

**Parameter**	**Visuospatial ability (*N* = 1,707)**			**Semantic knowledge (*N* = 1,707)**			**Episodic memory (*N* = 1,697)**		
	**MAP**	**95% HDI**		**MAP**	**95% HDI**		**MAP**	**95% HDI**	
**FIXED EFFECTS**									
Intercept, γ_00_	48.441	47.384	49.490	48.758	47.750	49.795	47.294	46.163	48.360
Linear slope (LS), γ_10_	−0.308	−0.357	−0.259	−0.021	−0.067	0.023	−0.198	−0.263	−0.131
Quadratic slope (QS), γ_20_	−0.142	−0.195	−0.088	−0.150	−0.200	−0.103	−0.121	−0.187	−0.051
Cohort, γ_01_	0.096	0.032	0.159	0.031	−0.031	0.096	0.248	0.180	0.317
Education, γ_02_	0.655	0.403	0.912	1.295	1.037	1.552	0.445	0.153	0.718
Sex, γ_03_	−1.444	−2.788	−0.037	2.649	1.367	3.961	1.835	0.417	3.283
Sample, γ_04_	−0.588	−1.237	0.070	0.461	−0.263	1.148	−0.959	−1.579	−0.316
LS × cohort, γ_11_	−0.001	−0.008	0.005	−0.006	−0.012	0.000	0.001	−0.006	0.008
QS × cohort, γ_21_	0.000	−0.002	0.002	0.002	0.000	0.004	0.002	0.000	0.005
LS × education, γ_12_	−0.001	−0.013	0.011	0.001	−0.010	0.013	0.006	−0.011	0.023
QS × education, γ_22_	−0.008	−0.021	0.005	0.001	−0.012	0.013	−0.013	−0.031	0.005
LS × sex, γ_13_	−0.009	−0.074	0.057	0.028	−0.031	0.092	−0.030	−0.121	0.059
QS × sex, γ_23_	0.029	−0.047	0.102	0.018	−0.050	0.085	−0.074	−0.168	0.026
Cohort × education, γ_05_	−0.007	−0.023	0.008	−0.019	−0.033	−0.003	−0.001	−0.019	0.016
Cohort × sex, γ_06_	−0.034	−0.124	0.057	0.059	−0.027	0.148	−0.116	−0.213	−0.018
Education × sex, γ_07_	−0.004	−0.363	0.338	−0.048	−0.388	0.298	0.344	−0.037	0.734
LS × cohort × education, γ_14_	−0.001	−0.002	0.001	0.000	−0.002	0.001	−0.001	−0.002	0.001
QS × cohort × education, γ_24_	0.000	0.000	0.001	0.000	0.000	0.001	0.001	0.000	0.001
LS × cohort × sex, γ_15_	0.000	−0.009	0.009	0.005	−0.003	0.013	−0.004	−0.014	0.006
QS × cohort × sex, γ_25_	−0.002	−0.005	0.001	0.000	−0.003	0.003	0.001	−0.003	0.005
LS × education × sex, γ_16_	−0.002	−0.019	0.015	−0.008	−0.024	0.008	0.002	−0.021	0.025
QS × education × sex, γ_26_	0.004	−0.014	0.022	−0.010	−0.028	0.007	0.011	−0.013	0.035
Cohort × education × sex, γ_08_	−0.001	−0.023	0.020	−0.034	−0.056	−0.013	−0.006	−0.032	0.018
LS × cohort × education × sex, γ_17_	0.000	−0.002	0.002	−0.001	−0.003	0.001	0.000	−0.002	0.003
QS × cohort × education × sex, γ_27_	0.000	−0.001	0.000	0.000	0.000	0.001	0.000	−0.001	0.001
**RANDOM EFFECTS**									
SD intercept, *u*_0_	6.887	6.619	7.152	7.213	6.917	7.527	5.703	5.403	6.004
SD linear slope, *u*_1_	0.042	0.011	0.094	0.143	0.108	0.170	0.075	0.026	0.141
Correlation (intercept, slope)	−0.514	−0.975	−0.095	0.490	0.372	0.641	0.494	0.213	0.964
Residual error	4.330	4.234	4.431	3.821	3.726	3.919	6.434	6.293	6.582

*Maximum a posteriori (MAP) estimates along with 95% highest density intervals (HDI). Cognitive scores are standardized to T-metric (mean = 50, SD = 10)*.

**Table 2:** Estimates of the focus parameter γ_12_ under different parametrizations of the alternative hypothesis.

**Parameter, *γ_12_***	**Prior σ**	**MAP**	**95% HDI**	**BF_01_**	**BF_10_**
Visuospatial ability	0.002	0.000	−0.004–0.004	1.072	0.932
	0.004	0.000	−0.010–0.009	1.547	0.646
	0.008	−0.001	−0.013–0.011	2.541	0.408
	3	−0.001	−0.013–0.011	–
Semantic knowledge	0.004	0.001	−0.006–0.007	1.226	0.816
	0.008	0.001	−0.009–0.010	1.771	0.565
	0.016	0.001	−0.010–0.012	2.822	0.354
	3	0.001	−0.010–0.013	–
Episodic memory	0.017	0.006	−0.010–0.019	1.755	0.570
	0.034	0.007	−0.011–0.022	3.193	0.313
	0.068	0.007	−0.011–0.022	6.156	0.162
	3	0.006	−0.011–0.023	–

“*Prior σ” is the variance of the prior, based on the estimates $\hat{\beta }$ from previous literature, and correspond to 1, 2, and 4 times $\hat{\beta }$. MAP the maximum a posteriori estimate; 95% HDI is the highest density interval that captures 95% of the posterior distribution; BF_01_ indicate the relative likelihood of the data under the null model vs. the alternative model, where the alternative model is parametrized in terms of Normal(0, σ); BF_10_ is the inverse of BF_01_. Weakly informed reference prior with σ = 3 is included for comparison of parameter estimates*.

### Linear decline, γ_10_

In line with previous literature in the field, the results show that visuospatial ability decline at about 0.31 standard deviations per decade (γ_10_ = −0.308, 95% HDI: −0.357 to −0.259), episodic memory decline about 0.20 standard deviations per decade (γ_10_ = −0.198; 95% HDI: −0.263 to −0.131), and semantic knowledge is relatively preserved even in old age (γ_10_ = −0.021, 95% HDI: −0.067 to 0.023).

### Acceleration of decline, γ_20_

Decline of all cognitive domains accelerate with advancing age, with semantic knowledge showing the most pronounced acceleration (γ_20_ = −0.150, 95% HDI: −0.200 to −0.103), followed by visuospatial ability (γ_20_ = −0.142, 95% HDI: −0.195 to −0.088) and episodic memory (γ_20_ = −0.121, 95% HDI: −0.187 to −0.051).

### Cohort effects, γ_01_

Performance on episodic memory increase about 0.25 standard deviations per decade (γ_01_ = 0.248, 95% HDI: 0.180 to 0.317). We could detect a marginal cohort-effect on visuospatial ability (γ_01_ = 0.096, 95% HDI: 0.032 to 0.159) but not for semantic knowledge (γ_01_ = 0.031, 95% HDI: −0.031 to 0.096). Note that these effects are statistically controlled for cohort differences in education.

### Education and level of cognitive function, γ_02_

We also replicate the finding that educational attainment has a modest association with level of visuospatial ability (γ_02_ = 0.655, 95% HDI: 0.403 to 0.912) and episodic memory (γ_02_ = 0.445; 95% HDI: 0.153 to 0.718), and a stronger association with semantic knowledge (γ_02_ = 1.295, 95% HDI: 1.037 to 1.552). This implies that, for example, every additional year of education above average is expected to increase visuospatial ability by 0.065 SD.

### Differential effect of education across cohort, γ_05_

We could not detect a differential effect of education across cohorts for visuospatial ability (γ_05_ = −0.007, 95% HDI: −0.023 to 0.008) and for episodic memory (γ_05_ = −0.001, 95% HDI: −0.019 to 0.016). However, our estimates suggest that—contrary to our expectation—the association between education and semantic knowledge might decrease for later birth cohorts (γ_05_ = −0.019, 95% HDI: −0.033 to 0.003). We emphasize that this is an exploratory analysis.

### Hypothesis testing: association between education and rate of decline, γ_12_

Bayes factors for H0: γ_12_ = 0 vs. H1: γ_12_ ~ Normal (0, σ), including sensitivity analyses and prior specification are presented in Table 2. Obtained MAP estimates are virtually identical under different priors; therefore, in the text we report parameter estimates using weakly informed priors.

The estimated effect of education on rate of cognitive decline is very small for visuospatial ability (γ_12_ = −0.001, 95% HDI: −0.013 to 0.011; BF_01_ = 1.1 to 2.5), semantic knowledge (γ_12_ = 0.001, 95% HDI: −0.010 to 0.013; BF_01_ = 1.2 to 2.8) and episodic memory alike (γ_12_ = 0.006, 95% HDI: −0.011 to 0.023; BF_01_ = 1.8 to 6.0). The Bayes factors (BF_01_) are all above 1, indicating some support for the null for all three outcomes. Inspection of the parameter estimates of γ_22_ also does not suggest that education alter the rate of acceleration of decline, γ_20_, for any of the cognitive outcomes.

The magnitude of the effect of education on linear decline (γ_12_) should be seen in light of the magnitude of the general linear rate of decline (γ_10_) as γ_12_ quantify the *change* in rate of decline depending on level of education. Similarly, the effect of education on acceleration of decline (γ_22_) should be seen in light of the general rate of acceleration of decline (γ_20_).

**Figure 1:**
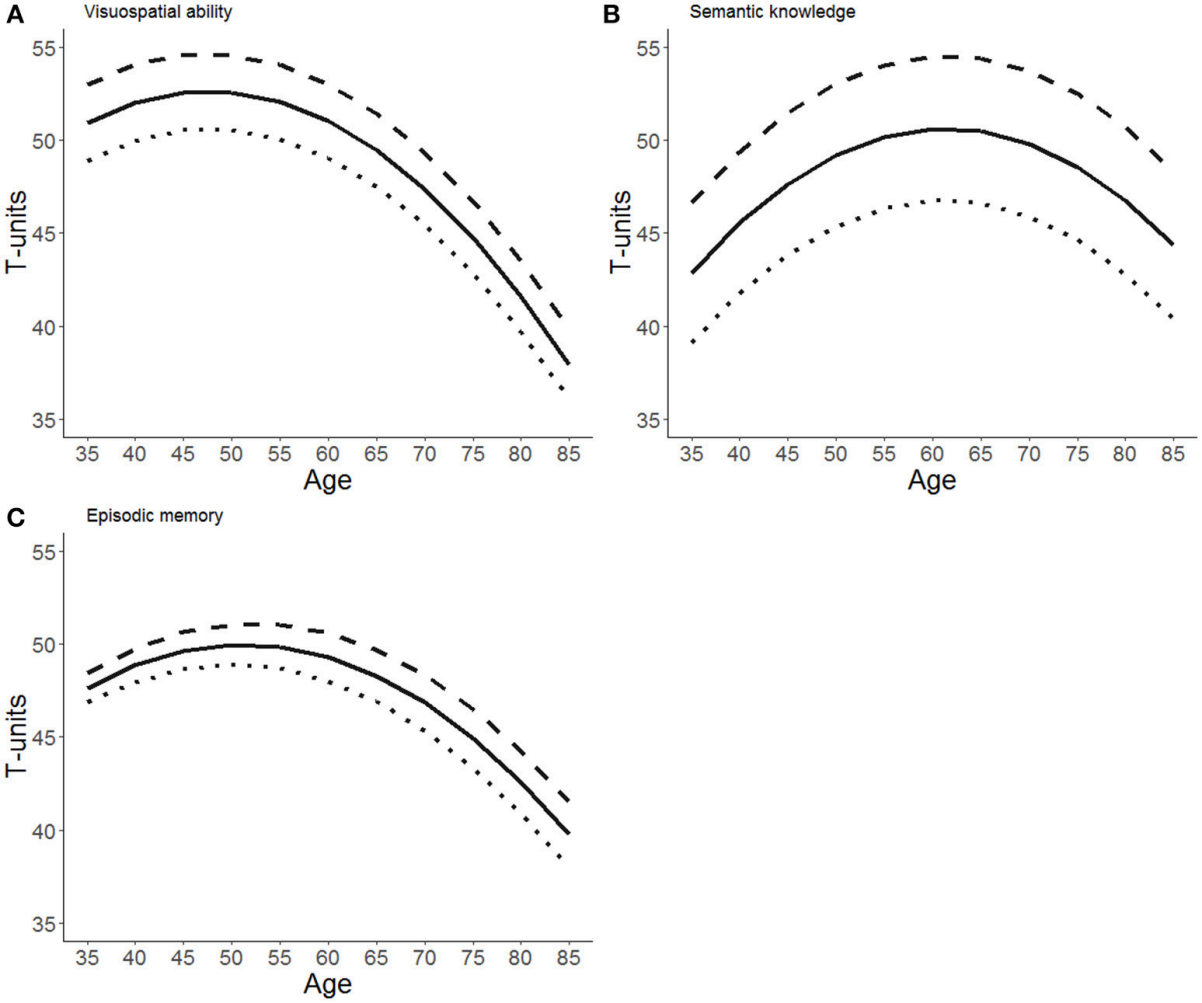
Model-implied growth trajectories for **(A)** visuospatial ability, **(B)** semantic knowledge, and **(C)** episodic memory. Trajectories apply to men born in 1935 with 3 years more than average education (dashed line), average education (solid line), and 3 years less than average education (dotted line).

Figure 1 illustrates the impact of education on level and slope for all three outcomes using estimates from the default, weakly informed model.

## Discussion

We replicate earlier findings that higher education is associated with higher cognitive function in a wide range of cognitive domains. We also find that visuospatial ability and episodic memory decline at a faster rate than semantic knowledge which is preserved even in old age. Further, we find that later birth cohorts perform better on episodic memory, even after statistically controlling for differences in years of education. More importantly, the MAP estimates of the focal parameters of interest γ_12_, while not exactly zero are at least very small indicating that each additional year of education does not alter the rate of decline in a substantively meaningful manner. This is illustrated in Figure 1 by parallel trajectories for different educational tiers. The parallel slopes are also validated by the Bayes factors BF_01_ > 1, indicating that the observed data are about as probable (in the case of visuospatial memory) to 6 times more probable (in the case of episodic memory) under the null hypothesis of no effect, than under a reasonably specified alternative hypothesis. However, our analysis is unable to clearly discriminate between the null hypothesis that education has *no* effect on decline in visuospatial ability or semantic knowledge, contrasted with the alternative hypothesis that education alters the rate of decline in these abilities with a magnitude reported in Gerstorf et al. (2011). As for episodic memory, we report Bayes factors ranging from 1.8 to 6.0, indicating (at best) substantial evidence in favor of the hypothesis that education do not alter the rate of cognitive decline, according to guidelines by Kass and Raftery (1995).

The Bayes factors need to be interpreted in light of the parametrization of alternative hypotheses. We consider the substantive hypotheses tested to be informative as well as plausible, as they are based on parameter estimates from previous studies. Thus, these estimates should reflect the current state of the field—if education has an effect on cognitive decline it is likely to be of a rather small magnitude. The estimates from Gerstorf et al. (2011) are all non-significant (i.e., the null hypothesis could not be rejected) and our findings are in line with this conclusion. Hülür et al. (2013) report a statistically significant effect of education on decline in episodic memory. Our findings—in contrast—indicate that the obtained results are still more likely under the null hypothesis of than under a reasonably specified alternative hypothesis. However, because the specified alternative hypotheses all posit effects of small magnitudes, our data is unable to clearly discriminate between the null and alternative hypotheses. Notably, alternative hypotheses positing larger effects yield stronger support for the null hypothesis, so somewhat small Bayes factors (e.g., Kass and Raftery, 1995) likely result from very conservative alternative hypotheses. Another interpretation of Bayes factors is that they quantify incremental *change* in belief about hypotheses. Seen this way, our data do not convey much *new* information, above and beyond previous findings. This is unsurprising, given that many large-scale studies have been done on the subject. Therefore, if one agrees with our prior specification, one shouldn't alter one's beliefs by any large degree after seeing these results—the prior is fairly well calibrated in relation to the likelihood (and there's not much difference between the prior and the posterior distribution for γ_12_). Thus, the evidence in the literature is converging toward no effect of education on cognitive decline, or at least, toward an effect so small so that it is unlikely to be of much theoretical or practical significance. This is consistent with recent systematic reviews (e.g., Lenehan et al., 2015) and earlier studies of education and cognitive decline (e.g., Zahodne et al., 2011).

One interpretation of our results therefore is that education is only important for understanding cognitive performance in older age because of the association between performance and education that is produced already in early development. These initial education-related differences in cognitive performance may result in individual differences in the age of onset of lost functional independence in late life. While there may be many factors affecting the rate of decline, we suggest that educational attainment is not one of them. Returning to the three variants of the cognitive reserve concept described in the introduction, this would support the model of passive reserve rather than active reserve or neural compensation (Barulli and Stern, 2013; Lenehan et al., 2015).

We were also interested in whether the effect of education on level of cognitive function depended on cohort. Because we had not identified any prior literature dealing with this substantive question, we were unable to specify a quantifiable alternative hypothesis. We therefore assessed this question by the summary estimates (MAP and 95% HDI) of the posterior distribution of the parameter γ_05_. For visuospatial ability and episodic memory, we found no evidence that the effect would vary across cohorts. However, the association between education and level of semantic knowledge seemed to *decrease* for later born cohorts, as indicated by the 95% HDI ranging from −0.033 to −0.003. This is contrary to our reasoning behind exploring this effect, which was that cognitive ability, in contrast to parental socio-economic status, may gain in importance for determining length of education in later born cohorts. We speculate that semantic knowledge may be an ability that is more strongly associated with parental socio-economic background than the more “fluid” visuospatial and episodic memory abilities. Therefore, semantic knowledge may have decreased in importance as a predictor educational attainment because socio-economic background has decreased in importance for access to higher education. Future confirmatory studies should further investigate the stability of education-cognition associations across historical times and cohorts.

A few limitations of the present work should be noted. One objection toward sharp-point (e.g., null) hypothesis testing is that all point hypotheses are known to be false *a priori*, and so testing a point-null is a futile exercise. While we can appreciate, and share, this concern, we work here under the assumption that insofar as the null hypothesis of invariance is worthy of rejection (e.g., evaluated using classical methods, as has been done in the past), it is at least worthy of consideration, and so should also be worthy of acceptance. When asking “is parameter γ different from 0?” one should be prepared to take “no” for an answer.

Another limitation is that the concept of reserve is meant to explain the discrepancy between observed symptoms of dementia and those predicted by observed brain pathology. As such, it is a concept that stems from pathological aging, whereas we apply it to healthy adults with no dementia diagnosis at the points of assessment. Another caveat is that we do not look at cognitive decline in very old age (ages 85+). It is possible that education alters the rate of decline in very old age, or among those with diagnosed dementia.

To conclude, we found that education is associated with level of cognitive function but unrelated to rate of decline in aging. We conclude that education is only a relevant variable for understanding cognitive performance in older age because of the association between performance and education that is formed in early development.

## Ethics statement

The data collection was approved at its inception by the Research Ethics Committee at Umeå University, Medical Faculty. No further ethical approvals pertaining to this particular study was required.

## Author contributions

RB and ML developed the study concept and study design. RB did the statistical analyses. RB drafted the manuscript and ML and JN critically revised the manuscript for intellectual content. All authors approved the final version of the manuscript before submission.

### Conflict of interest statement

The authors declare that the research was conducted in the absence of any commercial or financial relationships that could be construed as a potential conflict of interest.

## Footnotes

^1^According to Swedish law this data is not freely available but can be requested for specific research projects. The data analyzed for this study can be requested from the Steering Group of the Betula study; http://www.org.umu.se/betula/betula/access-betula-data/?languageId=1. This requires a data transfer agreement, which effectively transfers the confidentiality obligations of the institution at which the original research was conducted to the institution of the recipient of the data.
