# Supplementary material for: Education Does Not Affect Cognitive Decline in Aging: A Bayesian Assessment of the Association Between Education and Change in Cognitive Performance
Source: Front Psychol. 2018 Jul 6;9:1138. doi: 10.3389/fpsyg.2018.01138 (PMC6043857; doi:10.3389/fpsyg.2018.01138)
Supplement: Supplementary file 1 [file Table_1.DOCX]

| **Parameter** | **Visuospatial Ability** | | | **Semantic Knowledge** | | | **Episodic Memory** | | |
| --- | --- | --- | --- | --- | --- | --- | --- | --- | --- |
|  | **(*N* = 1707)** | | | **(*N* = 1707)** | | | **(*N* = 1697)** | | |
| *Fixed effects* | **MAP** | **95% HDI** | | **MAP** | **95% HDI** | | **MAP** | **95% HDI** | |
| Intercept, γ_00_ | 48.441 | 47.384 | 49.490 | 48.758 | 47.750 | 49.795 | 47.294 | 46.163 | 48.360 |
| Linear slope (LS), γ_10_ | -0.308 | -0.357 | -0.259 | -0.021 | -0.067 | 0.023 | -0.198 | -0.263 | -0.131 |
| Quadratic slope (QS), γ_20_ | -0.142 | -0.195 | -0.088 | -0.150 | -0.200 | -0.103 | -0.121 | -0.187 | -0.051 |
| Cohort, γ_01_ | 0.096 | 0.032 | 0.159 | 0.031 | -0.031 | 0.096 | 0.248 | 0.180 | 0.317 |
| Education, γ_02_ | 0.655 | 0.403 | 0.912 | 1.295 | 1.037 | 1.552 | 0.445 | 0.153 | 0.718 |
| Sex, γ_03_ | -1.444 | -2.788 | -0.037 | 2.649 | 1.367 | 3.961 | 1.835 | 0.417 | 3.283 |
| Sample, γ_04_ | -0.588 | -1.237 | 0.070 | 0.461 | -0.263 | 1.148 | -0.959 | -1.579 | -0.316 |
| LS x Cohort, γ_11_ | -0.001 | -0.008 | 0.005 | -0.006 | -0.012 | 0.000 | 0.001 | -0.006 | 0.008 |
| QS x Cohort, γ_21_ | 0.000 | -0.002 | 0.002 | 0.002 | 0.000 | 0.004 | 0.002 | 0.000 | 0.005 |
| LS x Education, γ_12_ | -0.001 | -0.013 | 0.011 | 0.001 | -0.010 | 0.013 | 0.006 | -0.011 | 0.023 |
| QS x Education, γ_22_ | -0.008 | -0.021 | 0.005 | 0.001 | -0.012 | 0.013 | -0.013 | -0.031 | 0.005 |
| LS x Sex, γ_13_ | -0.009 | -0.074 | 0.057 | 0.028 | -0.031 | 0.092 | -0.030 | -0.121 | 0.059 |
| QS x Sex, γ_23_ | 0.029 | -0.047 | 0.102 | 0.018 | -0.050 | 0.085 | -0.074 | -0.168 | 0.026 |
| Cohort x Education, γ_05_ | -0.007 | -0.023 | 0.008 | -0.019 | -0.033 | -0.003 | -0.001 | -0.019 | 0.016 |
| Cohort x Sex, γ_06_ | -0.034 | -0.124 | 0.057 | 0.059 | -0.027 | 0.148 | -0.116 | -0.213 | -0.018 |
| Education x Sex, γ_07_ | -0.004 | -0.363 | 0.338 | -0.048 | -0.388 | 0.298 | 0.344 | -0.037 | 0.734 |
| LS x Cohort x Education, γ_14_ | -0.001 | -0.002 | 0.001 | 0.000 | -0.002 | 0.001 | -0.001 | -0.002 | 0.001 |
| QS x Cohort x Education, γ_24_ | 0.000 | 0.000 | 0.001 | 0.000 | 0.000 | 0.001 | 0.001 | 0.000 | 0.001 |
| LS x Cohort x Sex, γ_15_ | 0.000 | -0.009 | 0.009 | 0.005 | -0.003 | 0.013 | -0.004 | -0.014 | 0.006 |
| QS x Cohort x Sex, γ_25_ | -0.002 | -0.005 | 0.001 | 0.000 | -0.003 | 0.003 | 0.001 | -0.003 | 0.005 |
| LS x Education x Sex, γ_16_ | -0.002 | -0.019 | 0.015 | -0.008 | -0.024 | 0.008 | 0.002 | -0.021 | 0.025 |
| QS x Education x Sex, γ_26_ | 0.004 | -0.014 | 0.022 | -0.010 | -0.028 | 0.007 | 0.011 | -0.013 | 0.035 |
| Cohort x Education x Sex, γ_08_ | -0.001 | -0.023 | 0.020 | -0.034 | -0.056 | -0.013 | -0.006 | -0.032 | 0.018 |
| LS x Cohort x Education x Sex, γ_17_ | 0.000 | -0.002 | 0.002 | -0.001 | -0.003 | 0.001 | 0.000 | -0.002 | 0.003 |
| QS x Cohort x Education x Sex, γ_27_ | 0.000 | -0.001 | 0.000 | 0.000 | 0.000 | 0.001 | 0.000 | -0.001 | 0.001 |
| *Random effects* |  |  |  |  |  |  |  |  |  |
| SD intercept, *u*_0_ | 6.887 | 6.619 | 7.152 | 7.213 | 6.917 | 7.527 | 5.703 | 5.403 | 6.004 |
| SD linear slope, *u*_1_ | 0.042 | 0.011 | 0.094 | 0.143 | 0.108 | 0.170 | 0.075 | 0.026 | 0.141 |
| Correlation (intercept, slope) | -0.514 | -0.975 | -0.095 | 0.490 | 0.372 | 0.641 | 0.494 | 0.213 | 0.964 |
| Residual error | 4.330 | 4.234 | 4.431 | 3.821 | 3.726 | 3.919 | 6.434 | 6.293 | 6.582 |

Table 1. Estimates from the full random effects model using weakly informative priors. Maximum a posteriori (MAP) estimates along with 95% highest density intervals (HDI). Cognitive scores are standardized to T-metric (mean = 50, SD = 10).
