# Supplementary material for: Education Does Not Affect Cognitive Decline in Aging: A Bayesian Assessment of the Association Between Education and Change in Cognitive Performance
Source: Front Psychol. 2018 Jul 6;9:1138. doi: 10.3389/fpsyg.2018.01138 (PMC6043857; doi:10.3389/fpsyg.2018.01138)
Supplement: Supplementary file 2 [file Table_2.DOCX]

| **Parameter, *γ_12_*** | **prior σ** | **MAP** | **95% HDI** | | | **BF_01_** | **BF_10_** |
| --- | --- | --- | --- | --- | --- | --- | --- |
| Visuospatial  ability | 0.002 | 0.000 | -0.004 | – | 0.004 | 1.072 | 0.932 |
|  | 0.004 | 0.000 | -0.010 | – | 0.009 | 1.547 | 0.646 |
|  | 0.008 | -0.001 | -0.013 | – | 0.011 | 2.541 | 0.408 |
|  | 3 | -0.001 | -0.013 | – | 0.011 | - | |
| Semantic knowledge | 0.004 | 0.001 | -0.006 | – | 0.007 | 1.226 | 0.816 |
|  | 0.008 | 0.001 | -0.009 | – | 0.010 | 1.771 | 0.565 |
|  | 0.016 | 0.001 | -0.010 | – | 0.012 | 2.822 | 0.354 |
|  | 3 | 0.001 | -0.010 | – | 0.013 | - | |
| Episodic  memory | 0.017 | 0.006 | -0.010 | – | 0.019 | 1.755 | 0.57 |
|  | 0.034 | 0.007 | -0.011 | – | 0.022 | 3.193 | 0.313 |
|  | 0.068 | 0.007 | -0.011 | – | 0.022 | 6.156 | 0.162 |
|  | 3 | 0.006 | -0.011 | – | 0.023 | - | |

Table 2. Estimates of the focus parameter γ_12_.“Outcome” designates cognitive test; “prior σ” is the variance of the prior, based on the estimates $\hat{\beta}$ from previous literature, and correspond to 1, 2 and 4 times $\hat{\beta}$; MAP the maximum a posteriori estimate; 95% HDI is the highest density interval that captures 95% of the posterior distribution; BF_01_ indicate the relative likelihood of the data under the null model vs. the alternative model, where the alternative model is parametrized in terms of Normal(0, σ); BF_10_ is the inverse of BF_01_. Weakly informed reference prior with σ = 3 is included for comparison of parameter estimates.
