# Supplementary material for: Education Does Not Affect Cognitive Decline in Aging: A Bayesian Assessment of the Association Between Education and Change in Cognitive Performance
Source: Front Psychol. 2018 Jul 6;9:1138. doi: 10.3389/fpsyg.2018.01138 (PMC6043857; doi:10.3389/fpsyg.2018.01138)
Supplement: Supplementary file 3 [file Presentation_1.PDF]

Berggren, Nilsson & Lövden, "Education does not affect cognitive decline in aging: A Bayesian assessment of the association between education and change in cognitive performance"

Supplement A - Model and prior specification

Model specification

The statistical model was estimated using the brms package in R. Using Wilkinson notation the model was specified as:

```
Y ~ 1 + age_1 + age_2 + cohort + education + sex + sample +
age_1:cohort + age_2:cohort + age_1:education + age_2:education +
age_1:sex + age_2:sex + cohort:education + cohort:sex +
education:sex + age_1:cohort:education + age_2:cohort:education +
age_1:cohort:sex + age_2:cohort:sex +
age_1:education:sex + age_2:education:sex +
cohort:education:sex + age_1:cohort:education:sex +
age_2:cohort:education:sex + (1 + age_1 | subject_id)
```

`Y` denotes the outcome variable (visuospatial ability, semantic knowledge, or episodic memory). Age was first mean-centered to a mean of 62.9 years of age, then decomposted into a linear term `age_1` and a quadratic term `age_2` using `R`'s `poly(x,2)` function. The age terms were then rescaled to their natural scale for ease of interpretation and prior formulation. `Cohort` and `education` was also mean-centered to year 1935 and 10.3 years of education, respectively. `Female` was coded as 0 for males, and 1 for females. `Sample` was coded as 0 for Sample 1 (S1) and as 1 for Sample 3 (S3). Cohort, education and female was allowed to interact with the linear and quadratic age effects. The random component includes random intercepts and random linear slopes for each subject.

Prior specification

We used weakly informative priors for all parameters except the age_1:education interaction term, which was subject to hypothesis testing using Bayes factors. How informative a actually prior is related to the scale of the posterior, so our default Normal(0,3) prior is more informative for main effects (e.g., the coefficient for age_1 and age_2) than for, say, three-way interactions (e.g. the education-by-cohort linear rate of decline, age_1:cohort:education). Thus, the Normal(0,3) prior performs much larger regularization for main effects than for 2-, 3- and 4-way interactions, potentially leading to under-estimates of the main effects. It should be noted that the effects reported in the article conform virtually identically to estimates obtained using classical methods, e.g. by R's `lmer()` function.

The full model was specified in `brms` using the following priors:

Model parameter	Parameter name	Prior
γ00	intercept	Normal(50, 20)
γ10	age_1	Normal(0,3)
γ20	age_2	Normal(0,3)
γ01	cohort	Normal(0,3)
γ02	education	Normal(0,3)
γ03	sex	Normal(0,3)
γ04	sampleS3	Normal(0,3)
γ11	age_1:cohort	Normal(0,3)
γ21	age_2:cohort	Normal(0,3)
γ12	age_1:education	*
γ22	age_2:education	Normal(0,3)
γ13	age_1:sex	Normal(0,3)
γ23	age_2:sex	Normal(0,3)
γ05	cohort:education	Normal(0,3)
γ06	cohort:sex	Normal(0,3)
γ07	education:sex	Normal(0,3)
γ14	age_1:cohort:education	Normal(0,3)
γ24	age_2:cohort:education	Normal(0,3)
γ15	age_1:cohort:sex	Normal(0,3)
γ25	age_2:cohort:sex	Normal(0,3)
γ16	age_1:education:sex	Normal(0,3)
γ26	age_2:education:sex	Normal(0,3)
γ08	cohort:education:sex	Normal(0,3)
γ17	age_1:cohort:education:sex	Normal(0,3)
γ27	age_2:cohort:education:sex	Normal(0,3)
u_0	SD intercept	Cauchy(0,10)
u_1	SD age_1	Cauchy(0,10)
ρ	COR intercept_slope	LKJ(1)

Fixed effects were given Normal(0,3) priors and random effects were given Half-cauchy(0,10) priors, restricted to positive values. Random effects correlation were given a uniform LKJ(1) prior.

Bayes factors are inherently a comparative measure between two explicitly formulated models, here H0: γ12 = 0 and H1: γ12 ~ Normal(0, σ). In Bayesian null hypothesis testing using Bayes Factors the value for σ explicitly capture the alternative hypothesis, H1, against which we test the null hypothesis, H0. One intuitive interpretation of H1 formulated this way is: "Small values are more probable than larger values, and there is a 95% probability that the true effect is between 0 ± 1.96*σ".

The age_1:education parameter γ12 was subject to hypothesis testing and was thus given an informed prior distribution γ12 ~ Normal(0, σ), with σ based on previous work by Gerstorf et al. (2011) and Hülür et al. (2013). This constitutes a conservative test of H0

because in order for the data to favor the null hypothesis, the posterior density for $\gamma 12$ has to be higher than the prior density for $\gamma 12$ at $\gamma 12 = 0$. With an already high prior density on $\gamma 12 = 0$, even small posterior shifts away from 0 would result in a posterior density with a lower value on $\gamma 12 = 0$, thus favoring H1. and 2) because we allow equal possibility of positive and negative values for $\gamma 12$.

Three different values for σ was used for each cognitive outcome, corresponding to alternative hypotheses positing larger effects on cognitive decline.

Model estimation

The model was estimated using R and the brms package. 4 chains were run for 2000 samples each, discarding the first 1000 samples. After sampling, convergence was assessed using the Gelman-Rubin Rhat statistic. The reported models all indicated convergence for all parameters, with rhat values below 1.01. For further validation that the model were set up properly, obtained parameter estimates was compared to classical estimates obtained from the lmer package.
